# Engaging Employers to Develop Healthy Workplaces: The WorkWell Initiative of Steps to a Healthier Washington in Thurston County

**Published:** 2009-03-15

**Authors:** Chris Hawkins, Mary Ann O'Garro, Kateri Wimsett

**Affiliations:** Thurston County Public Health and Social Services Department; Thurston County Public Health and Social Services Department, Olympia, Washington; Thurston County Public Health and Social Services Department, Olympia, Washington

## Abstract

**Background:**

The WorkWell initiative of Thurston County, Washington, established by Steps to a Healthier Washington in Thurston County (Thurston County Steps), focuses on recognizing and supporting local employers who make a commitment to address workforce health issues by implementing programs within their organizations to help adults reach *Healthy People 2010* objectives. This article reports on the WorkWell initiative and resulting WorkWell program.

**Context:**

The WorkWell initiative was developed to address the needs of private and public employers in Thurston County, Washington, to reduce the prevalence of chronic diseases through policy, practice, and environmental changes.

**Methods:**

Thurston County Steps recruited local employers to participate in advisory work groups to identify healthy workplace interventions that would be feasible for the employers and initiate a shift in organizational culture. The WorkWell initiative developed 2 distinct approaches — 1 for private sector (designation program) and another for public sector (action planning).

**Consequences:**

Twenty-six employers with approximately 4,700 employees were recognized with WorkWell Healthy Workplace designations for implementing changes that included encouraging stairwell use, providing low- or no-cost healthy meals for employees, and providing healthy foods at meetings. Four public agencies with approximately 4,400 employees have participated in an assessment and action planning process to help government employers focus their efforts and resources to support workforce health promotion.

**Interpretation:**

Unique partnerships between Thurston County Steps and other employers, private and public, demonstrate the important role employers can play in reducing chronic disease to improve a community's overall health.

## Background

In the United States, chronic disease accounts for 7 of the 10 leading causes of death (1) and affects the quality of life of 90 million Americans. Identifying effective and sustainable strategies to influence behavior at the local level is critical when addressing disability, health care costs, and the increasing prevalence of chronic diseases (2). Because most adults spend half of their waking hours at work, employers have a unique opportunity to create a work culture that improves the health of their workforce. Employees in optimal health are more likely to be on the job and performing well. They are also more likely to seek out jobs in these organizations and remain with an employer that values their health (3). Organizational support of health programs reduces employee turnover and has both fiscal and human resource benefits for employers (4,5).

In 2003, Washington State Department of Health (DOH) was 1 of 4 state health departments to be funded through a cooperative agreement from the Centers for Disease Control and Prevention (CDC). CDC's Steps Program provided federal funding for states, cities, and tribal entities to implement chronic disease prevention efforts to reduce the prevalence of obesity, diabetes, and asthma. Steps to a Healthier Washington in Thurston County (Thurston County Steps) was 1 of the 4 communities selected to work on this program in Washington State. The program focused on 3 related risk factors — physical inactivity, poor nutrition, and tobacco use — in Washington's health care industries, worksites, and schools. The Steps to a Healthier Washington program has used these federal funds in Washington's communities and across the domains to address asthma, nutrition, diabetes, tobacco use, and physical activity. The socioecologic model (6) provides a foundation for this multisector approach and addresses many behavioral determinants of health and many levels at which intervention can occur.

Thurston County Public Health, the Thurston County Chamber of Commerce, and various private and public employers used a model that empowers local communities to make sustainable changes through policy, collaborative leadership, and community mobilization, to develop the WorkWell initiative. The initiative encourages employers to adopt organizational policy, practice, and environmental change as a core strategy for workforce health promotion. Many employers, regardless of size or industry type, have found that addressing workforce health is not only desirable but also attainable. Organizational policies, a supportive social and built environment, and systemwide workplace practices help foster an organizational culture that facilitates healthy behaviors. Organizational policies, the formal rules that guide organizations, can exist in more forms than public policy or changes in law. They can change the way systems and institutions do business and can be voluntarily adopted by workplaces, schools, and other community-based organizations. Workplace practices are informal, unwritten expectations about the "way we do business." These kinds of practices reflect priorities and values of an employer, which then influence and shape workplace culture. A workplace culture that supports healthy lifestyles, where healthier choices have been made more accessible, can help change a person's overall health. Changes such as these typically have greater effect and sustainability (7,8).

## Context

Thurston County is in western Washington State and is the center for state government as well as a regional hub for medical services. The county has a population of 245,300, the seventh largest in the state. It is both urban and rural, with cities ranging from 660 to 44,800 residents (9). The county is a regional center for employment with approximately 129,200 jobs. Nearly 37,200 of these full- or part-time jobs are in federal, state, or local government (10). Thurston County Steps, led by the Thurston County Public Health and Social Services Department, aims to affect the county’s total population with emphasis on specific subpopulations by 1) implementing chronic disease prevention intervention programs and 2) working collaboratively with people who are influential in key aspects of the community (eg, neighborhood associations for built environment changes, health care providers for diabetes management, school districts for access to healthy food options). Thurston County Steps identified a need to develop a worksite health initiative to address public and private employers of all sizes and implement organizational policy, practice, and environmental changes that reduce the prevalence of chronic disease. The initiative attempted to reach small businesses like a graphic design firm and an office supply store (<50 employees), mid-sized commercial banks and local governments like the port and regional library district (up to 250 employees), and larger private businesses such as hospitals and state government agencies (>250 employees up to the largest at approximately 2,250).

## Methods

In 2004, Thurston County Steps recruited local employers from public and private organizations to identify workplace interventions that would be feasible for the employer and to improve employee health through workplace policy, practice, and environment changes. Employer-based advisory work groups were formed in 2005 to identify ways to raise awareness of health promotion strategies that could be used to improve employee health, decrease absenteeism, and reduce health care costs. The Thurston County Steps work groups' efforts led to the WorkWell initiative. Two different approaches were created to account for the differences in organizational culture, workforce composition, and resources among private and public employers. These approaches include specific evidence-based changes to the culture and environment of the workplace.

### Private sector WorkWell: Healthy Workplace Designation Program

With the help of a local private business owner, the Thurston County Steps program approached the Thurston County Chamber of Commerce about developing a joint venture aimed at private employers. The WorkWell Healthy Workplace Designation Program was developed during the first 6 months of 2006 to recognize the commitment and changes that employers made to support healthy eating and physical activity during work hours. The goal of the approach is to emphasize the many ways to bring about meaningful change within the workplace. Employers are invited to apply for recognition through the WorkWell Healthy Workplace Designation Program by documenting environmental and policy changes along with organizational commitment to health in the workplace from the previous year (8). Employers are recruited by direct marketing in e-mail, advertisement, and publicity on the Web sites of the chamber and the Thurston County Public Health and Social Services Department, in the chamber's monthly magazine, and through distributing program materials at chamber events.

The WorkWell program features 3 designation levels — bronze, silver, and gold. The bronze recognition is awarded to employers who demonstrate organizational support for changes to the work environment or culture that can affect healthy eating or physical activity. Examples of organizational support that qualify include expanding the organization's vision or strategic plan to incorporate employee health and fitness or starting a committee focusing on workforce health that allows employees in different job classes to assist in the design and implementation of plans for change. The silver recognition is awarded to employers who meet the criteria for bronze and have supported specific changes to the work environment that either 1) made healthier food choices available or more accessible to employees or 2) made engaging in physical activity during work hours an option or an easier choice for employees (11). The gold recognition is awarded to employers who have met the criteria for bronze and have supported changes that address both the healthy eating and physical activity criteria for silver. Types of qualifying efforts in the healthy eating category include creating a healthy food guideline for use when planning employer-sponsored events, having healthy food choices available in on-site vending or snack bars, and providing a refrigerator, microwave, and sink at no cost to employees. Examples of physical activity interventions include offering flexible break times and lunches on a rotating basis so employees can take advantage of physical activities, creating walking maps for destinations within 1 mile of the workplace, providing umbrellas for walking in rainy weather, and developing a policy that rewards employees who use a physically active way to travel to work. A review committee composed of local employers and Thurston chamber staff helped evaluate the applicants.

Recognized employers are given WorkWell decals that they can display on their entrance doors. The Thurston County Chamber of Commerce hosts a forum focused on awarding the designations, which in 2008 was attended by approximately 170 local employers. Employers are also honored in ways that communicate their unique approach to employee health and evidence-based strategies to their peers. Employers who receive the designation award are featured in an online video series promoted by the business community newspaper, a monthly magazine, and on the Thurston County Chamber of Commerce Web site.

### Public sector WorkWell: Action Planning for a Healthy Workforce

The WorkWell approach for public employers involves a guided self-assessment and action planning process called Action Planning for a Healthy Workforce. This process was designed to support employers who are ready to take a closer look at key organizational issues that affect employee health and fitness. Thurston County Steps developed the self-assessment tool and related promotional materials with an advisory committee consisting of managers and staff from local public sector (government) employers of varying sizes and workforce compositions.

The Action Planning for a Healthy Workforce process focuses on chronic disease areas that are consistent with the Steps Program’s target areas and were supported by the DOH evidence-based state plans (physical activity and nutrition [11], asthma [12] and tobacco use [13]). The self-assessment tool highlights 3 key components of workforce health promotion: 1) expression of leadership support and commitment to employee health (14-18), 2) expansion of written polices or formal practices in the organization (11,17-19), and 3) environmental assets and supports throughout the workplace (11).

The assessment and action planning process assumes that meaningful, long-term change must focus on both individual behavior (ie, employees) and organizational-level action (ie, worksites and employers). The belief that people shape, and are shaped by, their work environment is a key tenet of this model (Figure). Interested employers participate in a 2-stage process that provides decision makers with information to plan changes that would most benefit their organizations and workforces. The process starts with the facilitated completion of a self-assessment tool that identifies organizational strengths in relation to current health-related policy and practices. Employers are then invited to participate in a facilitated action planning process that identifies workforce health strategies designed to fit the employer's needs.

**Figure.**
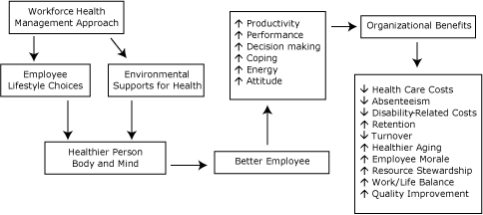
WorkWell program theory of change illustrates how a focus on workforce health may connect to key outcomes of interest to employers. Resources can be allocated to affect individual people or the environment to achieve desired outcomes. Employee Lifestyle Choices and Environmental Supports for Health are differentiated to highlight the program's interest in addressing whether healthier choices are present for selection in local workplaces. The arrows within the boxes indicate either increase or decrease in the listed item.

The Healthy Workforce assessment tool covers 4 main topics: 1) workforce health promotion, 2) physical activity at work, 3) healthy eating during work hours, and 4) air quality in the workplace. This assessment provides insight around characteristics of organizations that have been linked to successful changes in workforce health, including leadership support for workforce health and capacity for promoting change; a work environment organized or designed to make healthy choices easier; and culture of the organization, as demonstrated through written policies, formal practices, and communication. The assessment tool was developed by Thurston County Steps staff with expertise in program development, strategic planning, and organizational assessment.

The assessment is designed to be carried out by an assessment team consisting of the workplace's employees, thus involving the population affected by the possible actions and assuring greater accuracy and reliability in the data collection. The workforce perspectives included on the assessment team are senior leadership or policy makers, middle management, employee benefits, bargaining units, building maintenance or facilities management, internal wellness groups, and contract managers (eg, managing the services provided by food vendors, other worksite suppliers). Employers set their own timeline for the planning process; however, a 6- to 10-week schedule is recommended. Only 2 meetings of the full assessment team are needed to complete the self-assessment: a first for orientation and a second for scoring after the assessment data has been collected. A 2-person team of outreach and assessment staff from Thurston County Steps facilitate both the assessment and action planning phases and prepare the assessment report for the organization. The process was piloted with the Thurston County Public Health and Social Services Department, then became available for use with public-sector employers throughout the county.

In 2007, DOH became the first partner with the Thurston County Steps program to implement the assessment and action planning process to identify priority areas in their own agency. DOH chose this process as a way to address environmental and policy issues in the agency's overall health and productivity work. The agency followed the process described above, and Thurston County Steps reported results of the assessment back to DOH leadership. This report and the facilitation provided by Thurston County Steps helped DOH develop a baseline and prioritize work from its overall comprehensive plan. Thurston County Steps staff worked with DOH Health and Productivity Team members to develop an action plan based on identified agency priorities.

## Consequences

### Private sector WorkWell: Healthy Workplace Designation Program

In the first 2 years of the initiative in the private sector, 26 employers representing small and large workforces totaling approximately 4,700 employees, were awarded WorkWell designations (Table). Awardees received recognition for implementing changes that encouraged physical activity and healthy eating, such as making stairwells more appealing for physical activity, low-cost or no-cost healthy meals for employees, and healthy foods for meetings. Nineteen of the employers have been recognized with gold designation. From the first to the second year of the program, the number of employers who applied for recognition doubled (Table). Participating employers included banks, credit unions, social services agencies, a hospital, a casino, and several city and county government agencies.

The Thurston County Chamber of Commerce broadened its vision to encompass workplace health and began to feature WorkWell as one of the main programs for its members and other employers. The chamber signed a memorandum of agreement with Thurston County Public Health and Social Services Department to continue the collaboration around workplace health promotion. The chamber's Web site now promotes WorkWell and provides employers with examples of organizational support and healthy eating and physical activity options that they can implement to reach different designation levels. The WorkWell Healthy Workplace Designation Program was designed so that the Thurston County Chamber of Commerce became the lead entity for its implementation, further sustaining this valuable employer resource. The partnership will be sustained through funding provided by the US Department of Health and Human Services, Office on Women's Health Advancing System Improvements to Support Targets for *Healthy People 2010* (ASIST2010) program.

### Public sector WorkWell: Action Planning for a Healthy Workforce

Through September 2008, in addition to Washington State Department of Health and the Thurston County Public Health and Social Services Department, another state agency, a regional education service district, and a local government employer — together employing approximately 4,400 people — have completed the assessment, which demonstrates the versatility of this approach and its applicability to diverse public employers (Table). Three current employer partners are considering 18 workplace environment and policy changes.

Thurston County Steps has developed a sustainability plan for the public sector element of the WorkWell initiative. This plan includes exploring the interest among other public employers, particularly state agencies, in providing funding for the staff support provided by the Thurston County Public Health and Social Services Department. Thurston County Steps is also examining ways so that others can carry out the assessment and action planning process or repeat the process to reassess the workplace environment and policies as changes are being made. Thurston County Steps also revised the self-assessment tool based on comments and suggestions gathered during the first 2 years of its use.

## Interpretation

Steps successes nationwide have been built in part on creating and strengthening public-private partnerships. Thurston County Steps' partnerships in the WorkWell initiative demonstrate that collaborative approaches can be used to address community priorities and implement public health strategies that focus on the prevention and management of chronic disease. The benefits of partnerships are clear, but getting them established and operating in a sustainable manner that yields tangible results require careful consideration of diverse interests and needs of community partners. Unlike many federally funded community programs, Steps has a broad mandate to deal with risk factors that cut across many diseases and is expected to reach across multiple domains to create community-based solutions. This mandate has enabled Thurston County Steps to pull partners together to help create sustainable change. The initial leadership involvement from both private and public sector employers led to 2 distinct programs that have responded to the needs of the diverse worksites of Thurston County communities. The unique partnerships between the Thurston County Chamber of Commerce, several public sector employers, and the Thurston County Public Health and Social Services Department demonstrate the important role business and governmental employers can play in reducing chronic disease and improving a community's overall health.

## Figures and Tables

**Table.** Summary of WorkWell's Private and Public Sector Participants, Thurston County, Washington, 2004-2006

**Characteristic**	**Private Sector (Designation Program)**	**Public Sector (Action Planning)**
**No. of first-year (2006-2007) participating workplaces**	11	2
**No. of second-year (2007-2008) participating workplaces**	24	3
**No. of awards or actions**	3 Bronze level (commitment) 4 Silver level (commitment plus 1 change) 19 Gold level (commitment plus change in both healthy eating and physical activity)	18 Workplace environment and policy changes prioritized
**No. of employees at participating workplace with fewest employees**	2	Approximately 100
**No. of employees at participating workplace with most employees**	Approximately 2,200	1,700
**Total no. of employees at participating workplaces**	Approximately 4,700	Approximately 4,400
**Sustainability**	Thurston County Chamber of Commerce continues project	Plan in place; funding needed
